# Suppression of vascular endothelial growth factor expression in breast cancer cells by microRNA-125b-mediated attenuation of serum amyloid A activating factor-1 level

**DOI:** 10.18632/oncoscience.483

**Published:** 2019-07-02

**Authors:** Alpana Ray, Bimal K. Ray

**Affiliations:** ^1^ Department of Veterinary Pathobiology, University of Missouri, Columbia, Missouri

**Keywords:** Breast cancer, SAF-1, miR-125b, transcriptional regulation, VEGF expression

## Abstract

Increased level of an inflammation-responsive transcription factor called serum amyloid A-activating factor (SAF-1) has been linked to the pathogenesis in human breast cancer. SAF-1 is found to promote vascular endothelial growth factor (VEGF) expression in breast carcinoma cells and boost angiogenesis. In an effort to develop a cellular mechanism to control VEGF expression, we sought to limit SAF-1 activity in breast cancer cells. We report here several targets within the SAF-1 mRNA for binding of microRNA-125b (miR-125b) and we show that VEGF expression is reduced in breast cancer cells when SAF-1 level is reduced with the microRNA action. Within the 3' un-translated region (UTR) of SAF-1 transcript, we have identified four highly conserved miR-125b responsive elements. We show that these miR-125b binding sites mediate repression of SAF-1 by miR-125b. Ectopic expression of miR-125b in nonmetastatic and metastatic breast cancer cells repressed SAF-1-mediated activity on VEGF promoter function and inhibited cancer cell migration and invasion potentials *in vitro*. Together, these results suggest that termination of SAF-1 function by miR-125b could be developed as a potential anti-VEGF and anti-angiogenic agent, which has high clinical relevance.

## INTRODUCTION

Enhanced vascular endothelial growth factor (VEGF) expression is an important event in the growth and metastatic spread of breast cancer [1, 2]. VEGF, as an angiogenic factor, promotes new blood vessel formation. This process called angiogenesis plays a central role in the growth and progression of solid tumors by supplying nutrients, growth factors and oxygen and eventually assists in metastasis [3, 4]. Over-expression of VEGF in tumors primarily occurs due to transcriptional induction of *VEGF*. VEGF expression is induced by many factors, including cytokines, growth factors, hypoxia, differentiation, oncogenic transformation, which suggests for the presence of multiple routes to VEGF mRNA synthesis [5-7]. Since tumor cells are quite adept at acquiring new alternative processes to circumvent surrounding environmental pressures, identification of all regulatory pathways in tumor cells controlling VEGF expression is important. An inflammation-responsive transcription factor, called serum amyloid A-activating factor 1 (SAF-1) has been identified as a transcriptional regulator of VEGF during angiogenic switch in arthritic animals [8]. Later we have shown that SAF-1 is abundantly present in human breast cancer tissues and up-regulates VEGF expression in metastatic breast cancer cells [9].

High level of SAF-1 in breast cancer tissues and cells could also have additional implication for its role in cancer development and progression. For example, SAF-1 has been shown to regulate expression of several genes that play important role in cell invasion, cell proliferation and blood vessel remodeling. SAF-1 regulates several matrix metalloproteinases (MMPs) including MMP-1, MMP-9 and MMP-14 which are involved in extracellular matrix degradation and thus implicated in blood vessel growth and metastasis [10-12]. Human orthologue of SAF-1 regulates endothelial nitric-oxide synthase that plays quintessential role in remodeling and regulation of blood vessel [13] and ephrin-B2, a transmembrane ligand for Eph receptors that are implicated in angiogenic remodeling [14]. Human SAF-1 is shown to regulate c-myc proto-oncogene [15] and drive tumor specific expression of *PPARγ1* [16], overexpression of which correlates with breast cancer metastasis. Regulatory function of SAF-1 is mediated by its activity as a transcription factor. SAF-1 has a Cys2-His2-type zinc finger that is common to many other regulatory transcription factors such as Sp 1 and KLF family of transcription factors, just to name a few, and it is activated during inflammation [17-21].

Given the involvement of SAF-1 in regulating genes controlling tumor angiogenesis, cellular invasion, tumor cell proliferation and differentiation, and our previous finding of overexpression of SAF-1 in breast cancer [9], we aimed at developing a new approach to modulate SAF-1 by pharmacological treatment which would be an effective and promising method for cancer therapy. Indeed, the feasibility of this approach is validated by the observation of synthetic siRNA-mediated suppression of SAF-1 activity [9]. Availability of a natural RNA inhibitor would be more preferable for effective suppression of cellular SAF-1 gene. Here we report that a naturally occurring micro RNA, miR-125b, has the potential of regulating SAF-1 expression.

MicroRNAs (miRNAs) are endogenous small noncoding RNAs (20–23 nucleotides) and are known to regulate many biological processes, including development, cellular proliferation, apoptosis and differentiation [22]. In the cytoplasm, miRNAs negatively regulate gene expression at the post-transcriptional level by base pairing to the 3′ un-translated region (UTR) of target messenger RNAs and cause either mRNA degradation or translational arrest or both [22]. The miRNAs are found to be aberrantly expressed or mutated in cancer, suggesting that they may function as tumor suppressors or oncogenes depending on their expression status [reviewed in 23]. When miRNAs are over-expressed and inactivate tumor promoting molecules such as growth factors, they function as tumor suppressor [23]. Conversely, mutation in miRNAs may have an opposite effect. For example, when myc gene is translocated in B-cell leukaemia within the miR-142 locus, it causes mutation of this miRNA. Such a translocation results in an increased expression of myc and suppression of miR-142 expression. Thus, mutation of miR-142 has an oncogenic effect in B-cell leukaemia [23]. Among the different miRNA families, miR-125 family has been found to be associated with a variety of carcinomas [24]. In breast cancer, high level of expression of miR-125b has been shown to cause down-regulation of *ERBB2* (HER2) and *ERBB3* (HER3), and thereby suppression of tumor growth [25].

Since miR-125b has been shown to target VEGF expression in hepatocellular carcinoma [26], and our research has shown that VEGF expression is up-regulated by SAF-1 in breast cancer [9], we investigated whether SAF-1 could be a target of miR-125b. In the present study, we show that within the 3'UTR of human SAF-1 mRNA, four highly conserved miR-125b-responsive elements are present, in which three are adjacently present. Here, we also show that the repressive effect of miR-125b on SAF-1 is mostly mediated by these clustered miR125b-responsive elements. Furthermore, here we show that ectopic overexpression of miR-125b in breast cancer cells reduces *SAF-1* expression leading to decrease of VEGF level and consequential diminution of cancer cell migration and invasion. In correlation with previous microarray studies indicating decreased miR-125b expression in breast cancer tissues and miR-125b to be one of the most consistently deregulated miRNAs in breast cancer [27, 28], we report here that miR-125b is downregulated in breast cancer cells.

Since high level of SAF-1 in breast cancer is linked to angiogenesis, cancer cell proliferation and metastasis, a new approach to curb the activity of SAF-1 using microRNA may provide a new option in pharmacological treatment for breast cancer therapy.

## RESULTS

### Multiple miR-125b responsive elements in human SAF-1 mRNA

**Figure 1:**
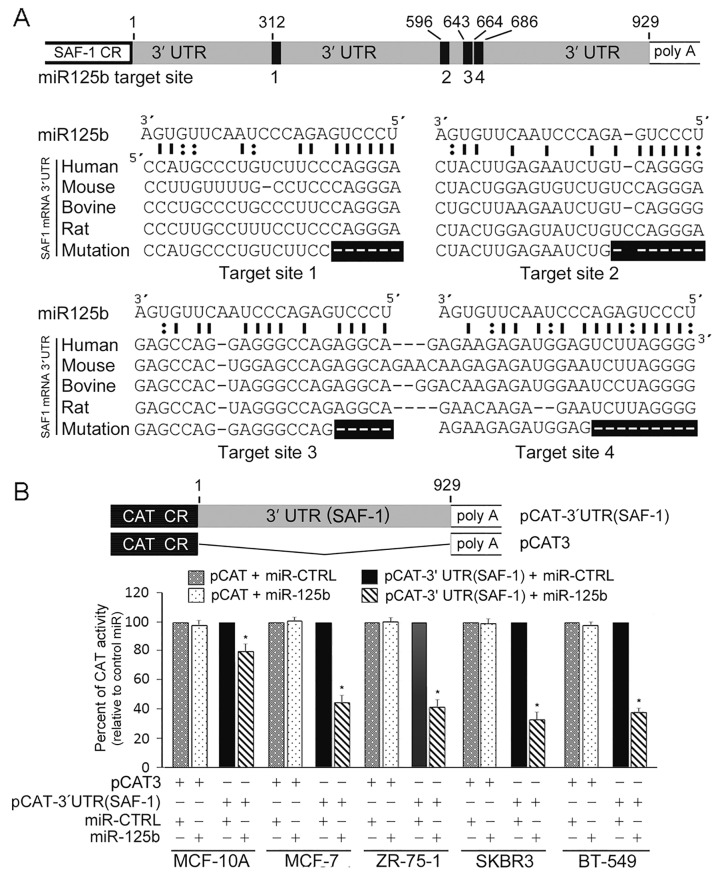
Human SAF-1 is a direct target of microRNA-125b (miR-125b) **(A)** Schematic representation of the structural features of SAF-1 mRNA shows SAF-1 coding region (SAF-1 CR) and poly A tail flanked by 929 nucleotide 3'untranslated region (UTR) containing four potential miR-125b binding sites (indicated by vertical black boxes and their relative nucleotide positions in the 3'UTR). Also shown are the nucleotide sequences of the predicted target sites and the seed sequences of miR-125b microRNA. Different species including human, mouse, bovine and rat contain very similar sequences at these sites. The deleted miR-125b-binding sites of SAF-1 3'UTR, indicated as horizontal black boxes, were constructed and inserted in mutant reporters which is described in Figure 2. **(B)** miR-125b inhibits SAF-1 3'UTR sequence-containing CAT reporter gene expression. Schematic representation shows map of pCAT-3'UTR(SAF-1) reporter plasmid where SAF-1 coding region was replaced by CAT coding region (CAT CR). MCF-10A (normal, nontumorigenic, immortalized breast cell), as well as MCF-7, ZR-75-1, SKBR3 and BT-549 breast cancer cells were transfected with pCAT-3'UTR(SAF-1) or control pCAT vector that lacks the SAF-1 3'UTR sequence. Following transfection, cells were incubated with 50 nM of pre-miR-125b or pre-miR-negative CTRL RNA, as indicated, for additional 48 h. CAT activity was determined in equivalent amount of cell extracts following a method as described in Materials and Methods. Relative CAT activity was determined by comparing the activities of miR-125b-transfected cells relative to the CAT activities in miR-control transfected cells. The results represent an average of three separate experiments. *, P < 0.05

Computer analysis to search for microRNA target recognition sites in the SAF-1 mRNA revealed four highly conserved sequence elements comprised of complementary core segment or the “seed” region of miR-125b binding element within the 929 nucleotides long 3'UTR (Figure 1A). The first miR-125b element (target site 1) is present about 300 nucleotides upstream of three clustered miR-125b elements (target sites 2-4). To determine if there is an interaction with miR-125b, the entire 3'-UTR of human *SAF-1* was ligated at the 3'end of the translational unit encoding chloramphenicol acetyl transferase (CAT) in the pCAT3 reporter plasmid. Expression of this reporter plasmid, pCAT-3'UTR (SAF1), in response to ectopic overexpression of miR-125b RNA was found to be substantially decreased in normal breast epithelial MCF-10A cells and in several breast cancer cells including ER positive MCF-7 and ZR-75-1 and ER-negative SKBR3 and BT-549 cells (Figure 1B). However, ectopic overexpression of scrambled miR RNA (miR-CTRL) did not decrease expression of the reporter plasmid, pCAT-3'UTR (SAF1), expression. Furthermore, pCAT3 reporter vector, that did not contain the 3'-UTR region of SAF-1, was not inhibited in response to overexpression of miR-125b RNA or scrambled RNA. Together these results suggested that the 3'-UTR of human SAF-1 mRNA is a potential target for repression by miR-125b.

### Mutational analysis of miR-125b-responsive elements

**Figure 2:**
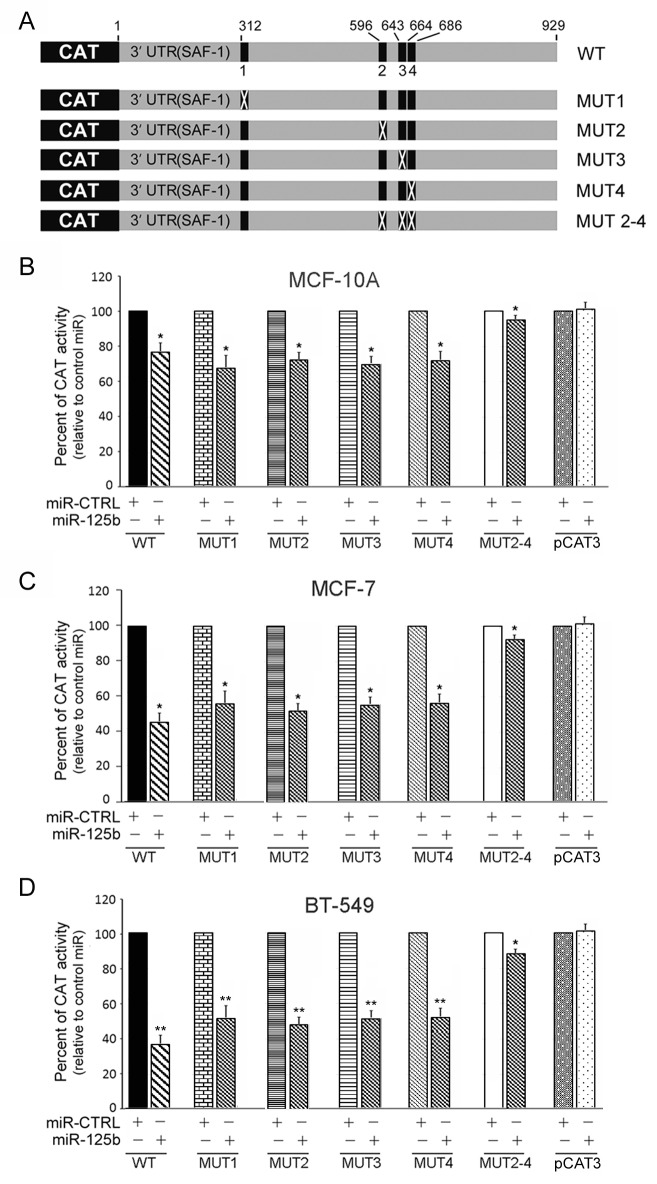
Mutational analysis of miR-125b-responsive elements (A) Physical map of the coding region of reporter gene CAT which is fused to the 929 nucleotide 3' untranslated region (UTR) of SAF-1 cDNA. The plasmid carrying this cassette is described in Figure 1B as pCAT-3'UTR(SAF-1) and is referred to as WT (for wild-type) in this Figure. This construct contains 4 potential target sites for miR-125b (represented by vertical black boxes numbered 1 through 4). Five different mutations were introduced at these sites including four single (MUT 1, MUT 2, MUT 3, MUT 4) and one multiple (MUT 2-4) mutations. Location of mutations is described in Figure 1A. (B) MCF-10A cells were transfected with either the wild-type (WT) pSAF1-3'UTR-CAT reporter, or mutant reporters (MUT1, MUT 2, MUT 3, MUT 4, or MUT 2-4. As a control, some cells were transfected with pCAT vector that lacks the SAF-1 3'UTR sequence. Following transfection, cells were incubated with 50 nM of pre-miR-125b or pre-miR-CTRL RNA, as indicated for additional 48 h and CAT activity was determined, as described in Figure 1B. The results represent an average of three separate experiments. *, P < 0.05 (C) MCF-7 cells were transfected with same set of reporter plasmids and further incubated with pre-miR-125b or pre-miR-CTRL RNA, as indicated in Figure 2B. Transfected cells were used for determining CAT expression, as mentioned above. The results represent an average of three separate experiments. *, P < 0.05. (D) BT-549 cells were transfected with same set of reporter plasmids and further incubated with pre-miR-125b or pre-miR-CTRL RNA, as indicated in Figure 2B. Transfected cells were used for determining CAT expression, as mentioned above. Results represent an average of three separate experiments. **, P < 0.01.

To determine the individual contribution among the four miR-125b target sites in SAF-1 mRNA 3'UTR, reporter plasmids containing deletion mutation of each of these four target sites, as indicated in Fig. 1A, were constructed. Four mutant plasmids with a single site mutated, referred to as MUT1 to MUT4, and one mutant with 3 sites mutated, MUT2-4 (Figure 2A), were used in the mutation analysis. These five reporter constructs with various mutated SAF-1 3'UTR were transfected into normal breast epithelial and breast cancer cells. Results are shown in Figures 2B-D. Deletion of a single element (MUT1, MUT2, MUT3 or MUT4) reduced but did not drastically eliminate miR-125b-mediated repression of pCAT-3'UTR (SAF1) reporter in MCF-10A or MCF-7 and BT-549 breast cancer cells (Figure 2B-D). However, when three clustered target sites of SAF-1 3'-UTR were mutated together in MUT2-4, there was almost no repressive effect of miR-125b. These results suggested that the clustered miR-125b target sites are the principal elements within the human SAF-1 3'-UTR that mediate repression by miR-125b.

### Impact of miR-125b overexpression on SAF-1 abundance in breast cancer cells

**Figure 3:**
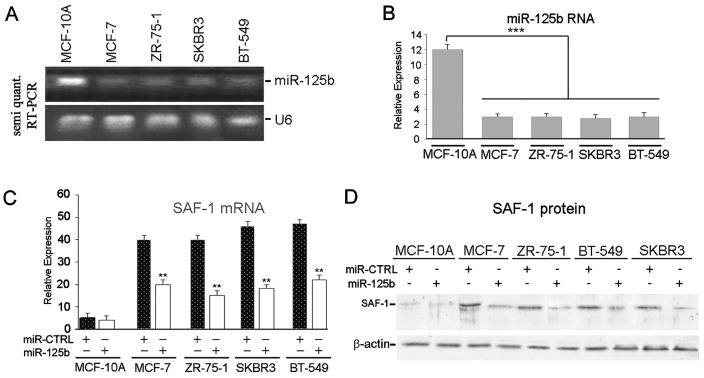
Ectopic expression of miR-125B suppresses SAF-1 mRNA and protein levels in breast cancer cells (A) miR-125b expression detection by end point semiquantitative RT-PCR. Total RNA isolated from MCF-10A and MCF-7, ZR-75-1, SKBR3 and BT-549 breast cancer cells were used for detection of miR-125b and U6 RNA transcripts using methods as described in Materials and Methods. (B) Quantitative RT-PCR analysis of miR-125b expression levels in MCF-10A, MCF-7, ZR-75-1, SKBR3 and BT-549 cells was accomplished by using primers specific for miR-125b and described in Materials and Methods. The results were normalized to the level of U6 in each sample and represent an average of three separate experiments. ***, P < 0.02. (C) Analysis of SAF-1 mRNA level in MCF-10A, MCF-7, ZR-75-1, SKBR3 and BT-549 cells following transfection with pre-miR-125b RNA or pre-miR-CTRL RNA. Quantitative RT-PCR methods, described in Materials and Methods, was used. Results represent an average of three independent experiments. **, P < 0.05. (D) SAF-1 protein level in MCF-10A, MCF-7, ZR-75-1, SKBR3 and BT-549 cells following transfection with miR-125b or miR-CTRL RNA was determined by Western blot analysis. Seventy μg protein samples from each cell extracts were fractionated and immunoblotted as described in Materials and Methods. The membrane was stripped and reprobed with β-actin antibody to confirm equal loading.

We noticed that in MCF-10A cells, the repressive effect of miR-125b is of much smaller magnitude as compared to that observed in the breast cancer cells (Figures 1 and 2). To investigate this phenomenon endogenous miR-125b expression levels in MCF-10A, MCF-7, ZR-75-1, SKBR3 and BT-549 cells were examined by semi-quantitative RT-PCR (Figure 3A) and qRT-PCR analysis (Figure 3B). These analyses clearly revealed that MCF-10A cells express miR-125b RNA at a much higher level than all breast cancer cells that were examined. Ectopic or forced overexpression of miR-125b RNA in these breast cancer cells substantially reduced SAF-1 mRNA (Figure 3C) and SAF-1 protein (Figure 3D) levels, which correlated with the 3' UTR-reporter assays as seen in Figures 1 and 2.

### Overexpression of miR-125b down-regulates SAF-1-mediated VEGF expression

**Figure 4:**
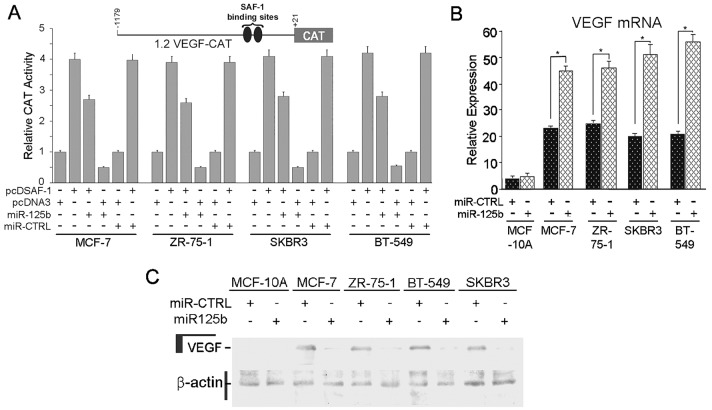
miR-125b suppresses SAF-1 activity that induces VEGF expression (A) MCF-7, ZR-75-1, SKBR3 and BT-549 cells were transfected with equal amounts (0.5 μg) of 1.2 VEGF CAT3 reporter whose expression is known to be induced by transcription factor SAF-1 (9, 35). In addition, some cells were co-transfected with 1.0 μg of either pcDSAF-1 expression plasmid, or pcDNA3 empty vector and/or pre-miR-125b RNA and miR-CTRL RNA, as indicated. Twenty four hours after transfection, cells were harvested and CAT activity in these cells was determined using an equivalent amount of cell extracts as described in Materials and Methods. Relative CAT activity was determined by comparing the activities in different transfected cells with that of pcDNA3-transfected cells and correcting for transfection efficiency (β-gal). Results represent an average of three independent experiments. Inset, schematic of CAT reporter plasmid containing human VEGF promoter sequence having two strong SAF-1-binding sites. (B) VEGF mRNA expression by qRT-PCR analysis in MCF-10A, MCF-7, ZR-75-1, SKBR3 and BT-549 cells following transfection with pre-miR-125b RNA or pre-miR-CTRL RNA. Results represent an average of three independent experiments. *, P < 0.05. (C) Western blot assay for VEGF protein level in MCF-10A, MCF-7, ZR-75-1, SKBR3 and BT-549 cells following transfection with pre-miR-125b or pre-miR-CTRL RNA in the same samples as shown panel B. β-actin level was measured to confirm equal loading of proteins.

As SAF-1 is a transcriptional inducer of VEGF [9], we examined the effect of miR-125b mediated repression of SAF-1 on VEGF expression. The transcription from 1.2VEGF-CAT reporter, which is dependent on the availability of SAF-1 [9], was down-regulated by miR-125b but not by control miRNA (Figure 4A). In correlation, ectopic expression of miR-125b RNA lowered VEGF mRNA (Figure 4B) and VEGF protein (Figure 4C) levels in the transfected breast cancer cells. In MCF-10A cells this response was minimum, which correlated with earlier results (Figure 3A-D).

### miR-125b impairs cell migration and invasive potentials of breast cancer cells

**Figure 5:**
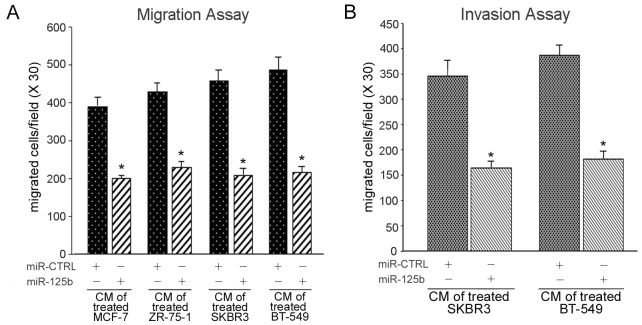
The effects of suppressing SAF-1 expression on breast cancer cell migration and invasion (A) Transwell migration assay. MCF-7, ZR-75-1, SKBR3 and BT-549 cells were transfected with pre-miR-125b or pre-miR-CTRL RNA, as indicated. Culture media from these transfected cells were collected 48 h later and used as conditioned medium (CM). Human umbilical vein endothelial cell lines, HUVEC-CS, were plated on the upper wells of transwell migration chambers and the culture media in the lower wells were fortified with these different CM preparations, as described in Materials and Methods. HUVEC-CS cells were allowed to migrate for 24 h and migrated cells were counted. Results represent an average of three independent experiments. *, P < 0.05. (B) Transwell invasion assay. SKBR3 and BT-549 cells were transfected with pre-miR-125b or pre-miR-CTRL RNA and plated on the upper wells of invasion chamber. Medium fortified with CM from the same cells, treated as indicated in panel A, was placed in the lower wells and cells were allowed to migrate for 24 h and cells invading through BioCoat Matrigel inserts were counted. Results represent an average of three independent experiments. *, P < 0.05.

All tumors must undergo angiogenesis to acquire nutrients for continued growth and metastatic spread. VEGF accounts for a cell migration of vascular endothelial cells and cellular invasion of tumor cells in cancer [29]. To verify if down-regulation of SAF-1 by miR-125b and subsequent reduction in the VEGF level has any measurable effect on angiogenic processes, we examined cellular migration of vascular endothelial cells (HUVEC). HUVEC were plated in trans-well chambers, incubated in presence of culture medium fortified with CM from breast cancer cells transfected with pre-mir-125b RNA, and observed for their ability to migrate through the membrane. Increase of miR-125b level resulted in ~ 50% lowered migration rate of cells as compared to scrambled pre-miR RNA transfected cells (Figure 5A). Invasiveness, requiring both cell motility and ability to advance through a solid and proteinaceous extracellular matrix like Matrigel, is thought to more directly assess cell metastatic potential. MCF-7 and ZR-75-1 breast cancer cells are regarded as noninvasive breast cancer cells, while SKBR3 and BT-549 cells exhibit invasive characteristics. Overexpression of miR-125b RNA, substantially reduced cellular invasive phenotype of both SKBR3 and BT-549 cells as compared to control pre-miR oligonucleotide (Figure 5B).

## DISCUSSION

Phosphorylation caused by cytokine- and growth factor-mediated signal transduction events plays a key role in the induction of transcriptional activity and DNA-binding activity of SAF-1 [19-21]. Increased SAF-1 activity is linked to pathogenesis of diseases including breast cancer [8-11, 30, 31]. While much is known about the activation of SAF-1, relatively little is known about its down-regulation or control in the cellular environment. Understanding of such a regulatory process would be useful in breast cancer therapy. Therefore, to identify a possible manner by which SAF-1 could be controlled intracellularly, we explored the possibility of microRNA mediated regulation of SAF-1. Our findings, described here, provide strong evidence that SAF-1 transcription factor is regulated by the microRNA miR-125b. The conclusion that miR-125b suppresses SAF-1 is supported by (i) demonstration of miR-125b elements in SAF-1 3'-UTR, mutation of which abolishes all regulatory effects of miR-125b on SAF-1 (Figures 1 and 2). We further show that (ii) overexpression of miR-125b causes reduction of SAF-1 mRNA level and protein expression in breast cancer cells (Figure 3), (iii) suppression of VEGF expression (Figure 4) and (iv) reduction of cell migration and (v) cell invasion in different types of breast cancer cells. (Figure 5)

The miR-125b target elements in the 3'UTR of SAF-1 mRNA are highly conserved across many species from human to rat (Figure 1) suggesting the possibility that these regulatory elements may play an important role in maintaining homeostasis in multiple species. We identified four elements among which three are adjacently present. Although, each of the miR-125b elements is similarly potent in mediating repression, simultaneous mutation of the three adjacent elements nearly abolished all repressive effects of miR-125b. Thus the three clustered elements appear to be the principal elements that mediate repression by miR-125b and necessary for efficient down-regulation of SAF-1.

The observed reduction in SAF-1 protein level in cancer cells following ectopic overexpression of miR-125b (Figure 3) can be reasonably attributed to miRNA effects on SAF-1 transcript stability and translation inhibition, as a previous report indicates that miR-125b can bifunctionally mediate both transcript decay and translational inhibition [32]. It will be interesting to find out whether relative positioning of the four miR-125b target elements in SAF-1 3'UTR would affect mRNA stability or translation or both.

Down regulation of miR-125b has been reported in primary breast cancers and metastatic breast tumors tissues [27, 33, 34]. We have also found much lower levels of miR-125b RNA in breast cancer cells in comparison to normal MCF-10A cells (Figure 3). Higher level of endogenous miR-125b RNA pool in MCF-10A cells can explain why SAF-1 mRNA and SAF-1 protein levels are so low in these cells as compared to those in breast cancer cells. This finding also explains the results in Figure 1B, 2C and 2D and suggests that the presence of already high level of endogenous miR-125b in MCF-10A cells most likely significantly suppresses most of SAF-1 level and thus further ectopic expression of miR-125b probably has less suppressive effect on SAF-1 in these cells. In contrary, in breast cancer cells, since endogenous level of miR-125b is low, there is very little suppression of SAF-1 level and thus further ectopic expression of miR-125b significantly suppresses SAF-1. This explains high level of inhibition of CAT activity by miR-125b in cancer cells which is seen Figures 1 and 2.

Our findings show that inhibition of endogenous SAF-1 activity by miR-125b action results in the significant inhibition of VEGF promoter function in the breast cancer cells (Figure 4) where the SAF-1 activity is much higher compared to normal cells [9]. This suggests a possible functional role of miR-125b in angiogenesis. Our previous studies have shown that in normal breast epithelial cells, MCF-10A, the CAT reporter expression in cells transfected with 1.2 VEGF-CAT reporter is very low and ectopic overexpression of SAF-1 in MCF-10A cells has minimal effect [35]. This suggested that in MCF-10A, high pool of endogenous miR-125b is able to sufficiently repress SAF-1 mRNA. In contrary, the breast cancer cells exhibit higher reduction of CAF activity under same condition suggesting lower level of endogenous miR-125b in these cells.

The microRNA mediated regulations are being increasingly implicated in tumorigenesis. Normally, miR-125b is expressed in many tissue types but its level is seen to be induced during neural differentiation, neurogenesis and during in vitro retinoic acid treatment of cells. However, the role of miR-125b in tumorigenesis is somewhat complex due to its diverse functions in different cell contexts [24]. In breast cancer and hepatocellular carcinoma, miR-125b expression becomes lower and such low expression is shown to be consequential for increased expressions of a number of cancer-associated genes that support tumorigenesis. These findings suggested miR-125b to be a tumor suppressor [25, 27, 28, 36-38]. However, expression of miR-125b is seen to be elevated in prostate cancer [39] and in a range of human leukemias [40-42] and ectopic expression of miR-125b is seen to favor stimulate cell proliferation and tumorigenicity [39] and cause highly invasive myeloid leukemia in the animals [43]. These diverse observations suggest that the role of miR-125b in cancer is dependent on the distinct pathophysiological conditions and the target molecules for the microRNA in different disease conditions. For therapeutic purposes, a careful selection of microRNA target may provide a desirable outcome.

In the present study, we observed that miR-125b overexpression reduces SAF-1 level. The effect of lower SAF-1 level, translated into a drop in VEGF expression and lowering of the tumorigenic potentials of breast cancer cells.

In summary, we report that SAF-1 is a direct target of miR-125b microRNA and reduced expression of miR-125b in breast cancer cells, partially accounts for upregulation of SAF-1 and VEGF in breast cancer. Regulation of miR-125b may have further implications in the complex events of tumorigenesis.

## METHODS

### Cell lines

MCF-10A, MCF-7, ZR-75-1, SKBR3, BT-549, and HUVEC-CS cells were obtained from American Type Culture Collection (ATCC), cultured and stored following ATCC protocol of authentication by STR analysis. The cells were maintained in Dulbecco modified Eagle medium (DMEM)-high glucose medium supplemented with 10% fetal bovine serum. For harvesting conditioned medium (CM) we followed a method that was described earlier [35]. Briefly, the cells were first grown in DMEM containing 7% FCS for 24 h. Next, the culture medium was replaced with DMEM containing 0.5% FCS, transfected with either miR-125b or miR-CTRL RNA and the cells were grown for an additional 48 h, after which the medium was collected, centrifuged at 1,000 x g, and stored at -80 °C for further use.

### Plasmid constructs, transfection and CAT reporter assay

The reporter plasmid 1.2VEGF-CAT3 was described earlier [9]. The pCAT-3'UTR (SAF1) was prepared by ligating 929 base pairs of human SAF-1 cDNA sequences representing entire 3'un-translated region (3'UTR) [44, 45] to the downstream of the coding sequence of chloramphenicol acetyl transferase (CAT) gene at the *Xba* I site in the pCAT3 promoter vector (Promega Corporation). The mutant reporters were constructed by megaprimer PCR amplification in which the individual miR-125b elements in the 3' UTR of SAF-1 were mutated using mutated PCR primers. Cells were transfected using lipofectamine 2000 (Invitrogen) following manufacturer's protocol. For CAT assay, cells were transfected by adding reporter plasmid DNA together with pSVβ-gal (Promega Corporation) plasmid DNA as described [11]. The pSVβ-gal DNA was used to monitor the efficiency of transfection and to normalize cell extracts used for CAT assay. To study the biological effects of miR-125b on breast cancer cells, cells were transfected with 50 nM of premiR-125b RNA (Invitrogen/Ambion; PM10148) or 50 nM of negative control (pre-miR-CTRL, Invitrogen/Ambion) molecules following the manufacturer's protocol.

### RNA isolation, semi-quantitative and quantitative RT-PCR analysis

The total RNA was isolated using a RNA isolation kit (Qiagen). Expression of miR-125b was assessed by semiquantitative RT-PCR, using a small RNA specific RT-PCR detection kit (Quantimir RT; Systems Biosciences) with the universal reverse primer and a human miR-125b specific forward primer (5'-TCCCTGAGTCCCTAACTTGT-3') by initial denaturation at 94 °C for 2 min, 22 cycles of 94 °C for 30s, 58 °C for 30s, 72 °C for 30s and a final extension for 5 min at 72 °C. The products were run in an agarose gel. For quantitative real-time PCR, the miRNA specific TaqMan MicroRNA Assay kits (Applied Biosystems) for miR-125b, SAF-1, VEGF and GAPDH were used by following manufacturer's protocol. Expression levels were normalized against two reference sequences, RNU44, RNU48 and/or GAPDH and relative changes were calculated. Experiments were run in triplicates.

### Western blot analysis

Cell extracts (70 μg) were fractionated using sodium dodecyl sulfate (SDS)-11% polyacrylamide gel electrophoresis (PAGE) and electroblotted onto PVDF membrane. The rabbit anti-VEGF and anti-β-actin antibodies were obtained from Santa Cruz Biotechnology. The rabbit anti-SAF-1 antibody, as described [10], was used at 1:5000 dilution. Bands were detected using a chemiluminescence detection kit (Amersham Biosciences).

### Cell migration and Invasion Assays

HUVEC migration assay was performed as described earlier (9, 35). Cells were trypsinized, pelleted and resuspended in medium without serum or growth factors. For migration assay, 1 x 10^5^ HUVEC cells in 0.5 ml medium were added onto 24-well transwell Boyden chamber with an 8-μm pore polycarbonate membrane. For invasion assay, 1 x 10^5^ SKBR3 and BT-549 breast cancer cells were placed onto the transwells which were precoated with 20 μg of Matrigel (BD Biosciences). For both cell migration and invasion assays, the transwells were then placed over bottom wells filled with conditioned medium (CM) of either miR-125b or miR-control (CTRL) RNA transfected breast cancer cells. After 24 h of incubation, HUVEC cells that did not migrate or cancer cells that did not invade through matrigel and remained on the insert top layers were removed with cotton swab and cells on the membrane lower surface, representing migrated and invaded cells, were fixed with 100% methanol for 2 min followed by staining with 1% toluidine blue solution. Inserts were washed several times before air drying; membranes were photographed and cell counts were determined after totaling five random fields. All assays were replicated three times.

### Statistics

To compare multiple sets of data, a one-way analysis of variance (ANOVA) with post hoc Fisher's least significant difference test was used. For paired data sets, a two-tailed t test was used. Values of P < 0.05 were considered to represent a significant difference.

## ABBREVIATIONS

SAF-1: serum amyloid A-activating factor-1
VEGF: vascular endothelial growth factor
miR-125b: micro RNA-125b
CTRL RNA: negative control RNA
UTR: un-translated region
siRNA: short interfering RNA
RT-PCR: reverse transcription polymerase chain reaction
qRT-PCR: quantitative RT-PCR
EMSA: electrophoretic mobility shift assay
PAGE: polyacrylamide gel electrophoresis
CAT: chloramphenicol acetyl transferase
